# A Hardware-Friendly Joint Denoising and Demosaicing System Based on Efficient FPGA Implementation

**DOI:** 10.3390/mi17010044

**Published:** 2025-12-29

**Authors:** Jiqing Wang, Xiang Wang, Yu Shen

**Affiliations:** School of Electronic and Information Engineering, Beihang University, Beijing 100191, China; wxiang@buaa.edu.cn (X.W.); shenyu45@buaa.edu.cn (Y.S.)

**Keywords:** partial convolution, demosaicing and denoising, reconfigurable architecture, unified acceleration computing platform, algorithm-hardware co-optimized

## Abstract

This paper designs a hardware-implementable joint denoising and demosaicing acceleration system. Firstly, a lightweight network architecture with multi-scale feature extraction based on partial convolution is proposed at the algorithm level. The partial convolution scheme can reduce the redundancy of filters and feature maps, thereby reducing memory accesses, and achieve excellent visual effects with a smaller model complexity. In addition, multi-scale extraction can expand the receptive field while reducing model parameters. Then, we apply separable convolution and partial convolution to reduce the parameters of the model. Compared with the standard convolutional solution, the parameters and MACs are reduced by 83.38% and 77.71%, respectively. Moreover, different networks bring different memory access and complex computing methods; thus, we introduce a unified and flexibly configurable hardware acceleration processing platform and implement it on the Xilinx Zynq UltraScale + FPGA board. Finally, compared with the state-of-the-art neural network solution on the Kodak24 set, the peak signal-to-noise ratio and the structural similarity index measure are approximately improved by 2.36dB and 0.0806, respectively, and the computing efficiency is improved by 2.09×. Furthermore, the hardware architecture supports multi-parallelism and can adapt to the different edge-embedded scenarios. Overall, the image processing task solution proposed in this paper has positive advantages in the joint denoising and demosaicing system.

## 1. Introduction

**Figure 1:**
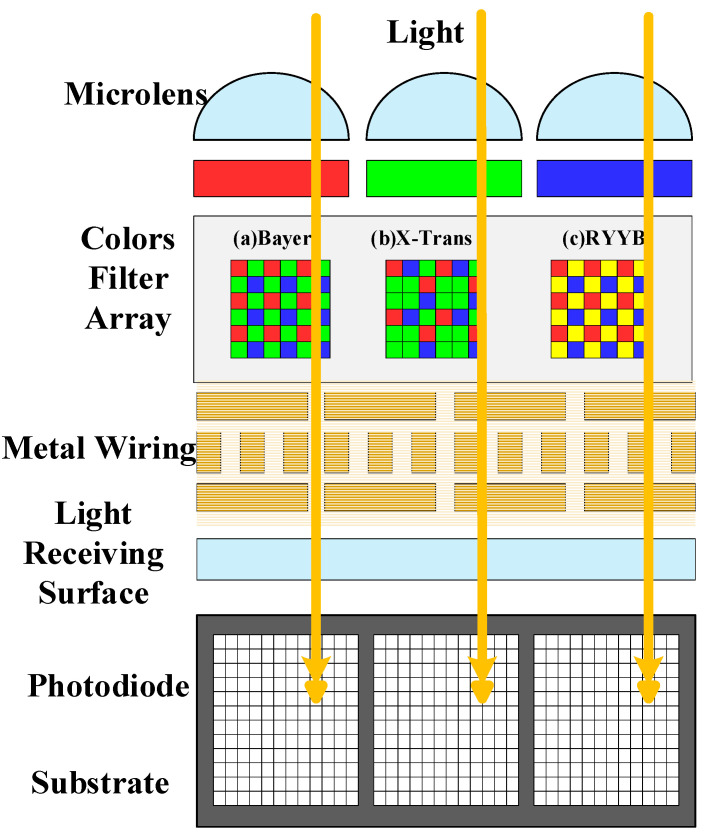
Basic CMOS structure and different CFAs.

Currently, the industry requirements for automotive cameras are becoming increasingly stringent. As the essential device in the perception layer of the advanced driver assistance system (ADAS), the vehicle camera is called the “eyes” of the autonomous driving system. It can capture high-level semantic information—such as color, texture, and text—that cannot be directly obtained by active sensors (e.g., radar and lidar). Table 1 shows that computing capability and the number of cameras are linearly related to the level of autonomous driving. Specifically, a complementary metal oxide semiconductor (CMOS) is the pre-processing unit of camera imaging, which is a semiconductor device that converts optical signals into electrical signals. In the CMOS image sensors, the most important component is the color filter array (CFA), as shown in Figure 1, which is divided into (Bayer/X-Trans/RYYB, etc.). It is widely used in digital cameras, smartphones, security monitoring, medical imaging, and other domains. In addition, the image post-processing unit in the image signal processor [1,2,3] (ISP) is also crucial. It determines whether the results can be delivered to the processing platform with high quality, low latency, and high reliability, especially in the multi-camera and distributed smart car architecture.

**Figure 2:**
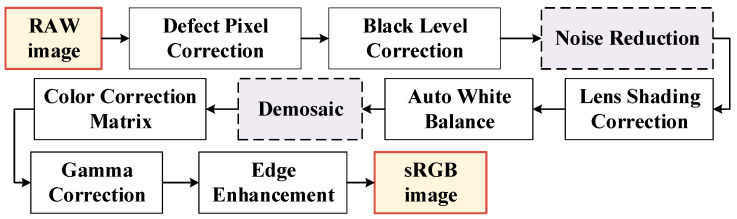
ISP pipeline.

The CMOS image sensors typically output RAW data, which must be processed by the ISP to carry out fundamental operations such as demosaicing. A typical ISP processing pipeline is depicted in Figure 2. As an essential stage in the imaging process, the demosaicing module reconstructs a full-resolution sRGB image from the sampled sensor data and significantly impacts the quality of the final output [3,5,6,7]. More specifically, on the one hand, most color digital cameras and smartphones use image sensors equipped with a CFA that captures only one color component per pixel [1,3,8,9,10]. Another challenge is dark current noise, which distorts the captured raw data and causes various problems such as false color and zipper noise [11,12].

Until now, deep convolutional neural networks (CNNs) have been the most popular technology in computer vision and image processing [13,14,15,16]. In order to overcome the shortcomings of traditional schemes, the research trend is to achieve full-resolution image restoration and reconstruction through joint processing through neural networks [17]. For example, Tesla’s autopilot system aims to develop a vision processing solution based on cameras, using massive data in reality to train end-to-end neural network models, which can deal with various driving scenarios with reliability and safety. In this framework, the artificial intelligence-based image signal processor (AI-ISP) is an essential and indispensable component for achieving autonomous driving.

Most joint processing solutions are designed based on software solutions [3,6,18,19,20,21,22]. Although the indicators have been significantly improved, practical deployment of image signal processors based on AI still requires hardware-implementable solutions. Field-Programmable Gate Array (FPGA) hardware acceleration represents a prominent research focus and is regarded as one of the most promising applications in the future. It is widely recognized that FPGAs offer the most efficient means for early-stage hardware verification.

Since most existing network architectures are over-parameterized and struggle to meet real-time performance on edge devices, it is crucial to optimize CNN structures for deployment on resource-constrained, low-power embedded platforms with flexible deployment requirements. The computational efficiency and accuracy of CNN are equally important, so there is a lot of work devoted to improving efficiency. The more popular grouped convolutions and depth-separable convolutions (consisting of depthwise and pointwise convolutions) [23,24,25]. Previously, these works reduced parameters and floating-point operations by considering the redundancy of filters. Conversely, the running speed of the model did not increase accordingly due to the low computational efficiency caused by frequent memory access. To address this issue, this paper introduces partial convolution (PConv) [26] and proposes a lightweight Partial-CNN backbone network (LJDD-Net) to accelerate image processing tasks such as demosaicing and denoising, with potential extensions to other low-level vision applications such as super-resolution and so on. To summarize, our contributions are as follows:

- At the algorithm level, a hardware-inspired and multi-stage network structure (LJDD-Net) is proposed, which achieves excellent restoration quality with lower model complexity and tends to improve latency and efficiency. Compared with the standard convolutional solution, the parameters and MACs are reduced by 83.38% and 77.71%, respectively.
- Based on algorithm–hardware co-design, a unified and flexible mechanism is employed. The computing unit adopts a fully pipelined dataflow architecture with a scalable hardware interface, which effectively addresses resource constraints across different platforms. In addition, a padding mechanism based on address mapping is proposed to achieve zero resource overhead.
- The hardware architecture with multiple levels of parallelism, implemented on a Xilinx development board, achieves a 2.09× improvement in computational efficiency over the state-of-the-art method [27]. Compared with the two parallelization schemes, the proposed hardware accelerator achieves energy-efficiency improvements of 72.56× and 24.28× on the CPU platform, and 85.39× and 28.58× on the GPU platform, respectively. Moreover, the design demonstrates the advantages of high quality, low cost, and high performance, while maintaining a balanced trade-off between accuracy and computational efficiency, as verified in subsequent experiments.

The rest of the paper is organized as follows. In Section 2, we review the related works about demosaicing and denoising. In Section 3, we propose the multi-stage network structure, and Section 4 introduces the designed hardware acceleration structure and optimization scheme. In Section 5, we show the experimental comparison and ablation analysis results. Finally, the paper is concluded in Section 6.

## 2. Related Work and Motivation

In this section, we briefly introduce the related works about various methods for demosaicing and denoising (DM&DN). Presently, there are mainly traditional algorithms, deep learning solutions, hardware-based methods, etc. Thus, we summarize the related works of multi-stage processing methods in deep learning and hardware FPGA-based solutions. In addition, we compare the number of parameters and computing time of different types of convolutions under the test conditions.

### 2.1. Multi-Stage Methods

With the rapid development of deep learning, DM&DN methods based on convolutional neural networks have been widely studied. Michael Gharbi et al. first introduced a multi-stage deep learning method in 2016 [28] to study the DM&DN and denoising task, and the model network structure was an order of magnitude faster than the previous state-of-the-art technology. Subsequently, Wang et al. [7] proposed a DM&DN network structure based on UNet++, which replaced the previous downsampling optimization with Gaussian smoothing. To avoid the defects of traditional methods, such as moiré and zipper noise, Khadidos et al. [29] proposed a texture network along with a deep smooth texture residual neural network. Although the above methods are significantly optimized compared to traditional algorithms, these single-stage neural network processing methods are rarely deployed on embedded devices. In addition, there is a multi-stage training neural network to further improve the effect of the model. Cui et al. [30] used a three-stage convolutional neural network structure to perform DM&DN. In addition, there are methods that combine traditional algorithms and deep neural networks [12,31,32,33]. Overall, these are solutions implemented in software [18,34,35,36], and there are no specific solutions for hardware implementation.

### 2.2. Design Challenges in DM&DN Hardware Solutions

In hardware implementations of DM&DN tasks, traditional algorithms [10,37,38,39] such as bilinear interpolation [40] and Adams–Hamilton [41] adaptive interpolation [42] are commonly applied. However, they often require extensive hardware debugging and leave significant space for post-deployment optimization. Ultimately, the quality of the final image largely depends on the technical expertise of the developers. To address this limitation, recent research has explored AI-based implementations for the full or partial pipeline of AI-ISP systems [43,44,45,46,47], such as the DM&DN task in this article. It is undeniable that the above research has achieved remarkable results in the processing of traditional difficult ISP scenarios.

However, the high computational requirements of AI-ISP limit its feasibility for extensive engineering applications. How to reduce computational complexity while improving accuracy has become a crucial challenge in both academia and industry. Guan et al. [27] first introduced deformable convolution in the joint DM&DN task. However, the balance between computational efficiency and accuracy was not considered, and there are enormous challenges in the hardware implementation. Thus, this paper proposes a faster and lighter network architecture for the DM&DN tasks, and proposes a unified hardware acceleration solution to support different operators.

### 2.3. Exploring Various Convolution Operations

To design faster neural networks, we take standard convolution as an example to analyze its time and space complexity. The computational complexity (floating-point operations, FLOPs) is defined as

(1) $$Time∼O(\sum_{l=1}^{D}m_{l}^{2}\times k_{l}^{2}\times c_{in}\times c_{out})$$

The complexity of the computational space (the amount of memory access) can be synthesized by

(2) $$Space∼O(\sum_{l=1}^{D}k_{l}^{2}\times c_{in}\times c_{out}+\sum_{l=1}^{D}{m_{l}}^{2}\times (c_{out}+c_{in}))$$

In (1) and (2), $m_{l}$ represents the size of the feature map, $c_{in},c_{out}$ represents the channel dimension of input and output, and $k_{l}$ represents the size of the convolution kernel.

**Figure 3:**
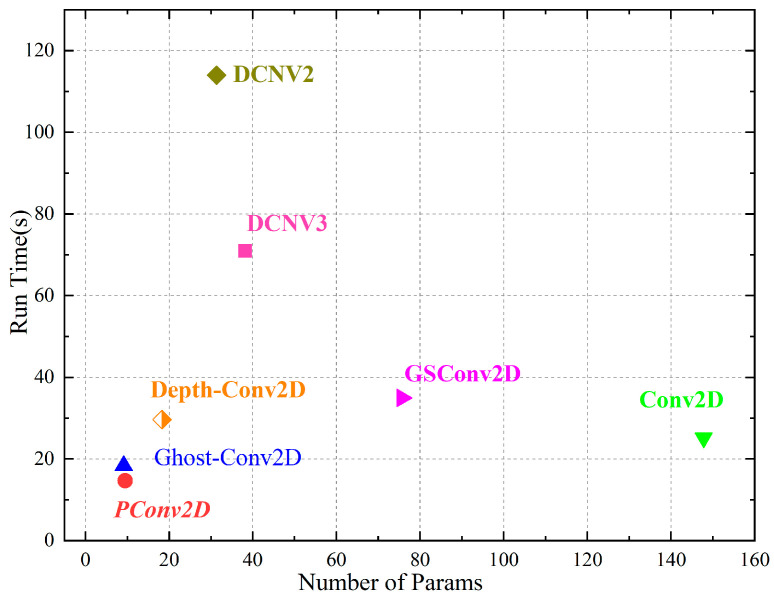
Comparison of the number of parameters and running time of different convolution types. (Conv2D: standard convolution; Depth-Conv2D: depth-separable convolution; DCNv2 and DCNV3 are two versions of deformable convolution; Pconv: partial convolution; GhostConv: ghost convolution; GSconv: mixed convolution of SC, DSC, and shuffle).

As shown in Figure 3, it can be seen that the partial convolution (PConv) [26] processing method is superior to other convolution schemes in terms of computational latency and parameters. That is to say, the throughput of partial convolution is the optimal performance indicator, and it is cost-effective when deployed on edge-embedded devices. The PConv can extract spatial features more efficiently by simultaneously reducing redundant computations and memory accesses. This approach can reduce FLOPs while maintaining high FLOPS (floating-point operations per second), reduce memory latency, and also improve frames per second, where latency is defined as

(3) $$Latency\;=\;\frac{FLOPs}{FLOPS}$$

It is a competitive convolution operation that exploits redundancy in feature maps to systematically apply regular convolutions to certain input channels without affecting the remaining input channels. Finally, the channel output is performed through the fusion module, the FLOPs of the PConv are only 1/16 compared to the standard convolution, and the amount of memory access in the (5) is

(4) $$h\times w\times k^{2}\times c_{p}^{2}$$

(5) $$h\times w\times 2c_{p}+k^{2}\times c_{p}^{2}\approx h\times w\times 2c_{p}$$

**Figure 4:**
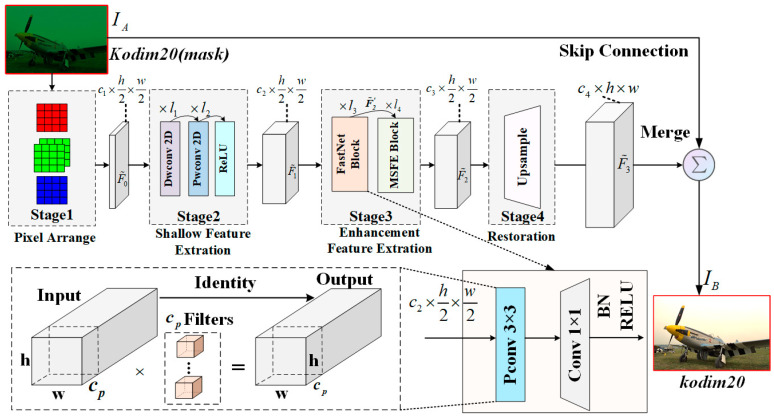
Overall architecture of our LJDD-Net. In the third stage, we facilitate the FastNet block to replace the conventional 16-layer backbone structure [28]. Compared with the previous operation, multiplication and addition are from 2.34 G to 88.23 M (the test vector is 128 × 128). Four batch normalization layers are inserted into the multi-scale feature extraction module (MSFE) and can be merged with convolution layers to reduce the subsequent computational complexity on the hardware. Additionally, since the DM&DN task is sensitive to image texture details, the pooling operator is removed, and the “same” convolution mode is applied in the backbone model structure to ensure the extraction of edge texture features. All operators have a stride of 1, except for the pixel alignment stage, where the stride is 2.

It can be seen that it is approximately equal to the memory access of only the input and output channels (in this paper, $c_{p}=c/4$, as shown in Figure 4).

## 3. Network Design Incorporating Partial Convolution

In this section, we explain the reconstruction problem of RAW-sRGB in Section 3.1. Subsequently, we propose LJDD-Net to solve the problem of reconstruction. Section 3.2 and Section 3.3 introduce the detailed architecture of the overall network. Finally, Section 3.4 describes the number of model parameters and the size of feature maps.

### 3.1. Definition of RAW–sRGB Pairs

In a single-sensor camera equipped with a color CFA, each pixel records only one of the three-color components (R, G, or B). Considering the CFA as shown in Figure 1, the CFA sampling process can be modeled by applying a corresponding color mask to the RGB image. The sRGB is generated through a mask and the $M_{CFA}$ mapping matrix can be calculated as

(6) $$M_{CFA}=\left[\begin{array}{l} M_{R}.*R(i,j)\\ \;\;\;\;(M_{GR}+M_{GB}).*G(i,j)\\ \;\;\;\;\;\;\;\;\;\;\;\;\;\;\;\;\;\;\;\;\;\;\;\;\;M_{B}.*B(i,j) \end{array}\right]$$

where, $\Omega =[1,2,\cdot \cdot \cdot ,N_{1}]\times [1,2,\cdot \cdot \cdot ,N_{2}]$ R(i,j)∈Ω; G(i,j)∈Ω B(i,j)∈Ω; R,G,B denotes the red, green, and blue channel matrix respectively; and $M_{R},\;M_{GR},\;M_{GB},\;M_{B}$ is the CFA mask. “*” denotes the element-wise multiplication of two scalar matrices of the same dimension. The mask matrix is defined as

$$M_{R}(i,j)=\left\{\begin{array}{l} 1,\;if(i,j)\in \Omega_{R}\\ 0,\;if(i,j)\notin \Omega_{R}\;\;\; \end{array}\right.M_{GR}(i,j)=\left\{\begin{array}{l} 1,\;if(i,j)\in \Omega_{GR}\\ 0,\;if(i,j)\notin \Omega_{GR}\;\;\; \end{array}\right.\;M_{B}(i,j)=\left\{\begin{array}{l} 1,\;if(i,j)\in \Omega_{B}\\ 0,\;if(i,j)\notin \Omega_{B}\;\;\; \end{array}\right.M_{GB}(i,j)=\left\{\begin{array}{l} 1,\;if(i,j)\in \Omega_{GB}\\ 0,\;if(i,j)\notin \Omega_{GB}\;\;\; \end{array}\right.$$

where, $\Omega_{R},\Omega_{GR},\Omega_{RG},\Omega_{B}\subseteq \Omega$ are disjoint pixel sets recording red, green, and blue values, respectively, and $\Omega_{R}\cup \Omega_{GR}\cup \Omega_{RG}\cup \Omega_{B}=\Omega$. The green pixel set $\Omega_{G}$ consists of $\Omega_{GR}$ and $\Omega_{RG}$, $\Omega_{GR}\;\cup \;\Omega_{RG}\;=\Omega_{G}$, $\Omega_{GR}\cap \;\Omega_{GB}=\emptyset$. The green mask and non-green mask are defined as

(7) $$M_{G}=M_{GR}+M_{GB},M_{IG}=M_{R}+M_{B}$$

The complete sRGB matrix is represented as

(8) $$I_{B}=M_{R}.*R+M_{GR}.*G+M_{GB}.*G+M_{B}.*B$$

A pairwise raw-to-raw calibration to map image $I_{A}$ to image $I_{B}$ can be expressed as

(9) $${\hat{I}}_{B}=g(M_{CFA}\;\cdot \;\phi (r(I_{A}(x)))+\sigma (x))$$

where r(·) and g(·) are reshaping functions that represent images as 3×n (*n* = the total number of pixels in each image) and h×w×3, respectively. φ(·) is a kernel function and σ(x) is dark current noise.

### 3.2. Proposed Method

**Figure 5:**
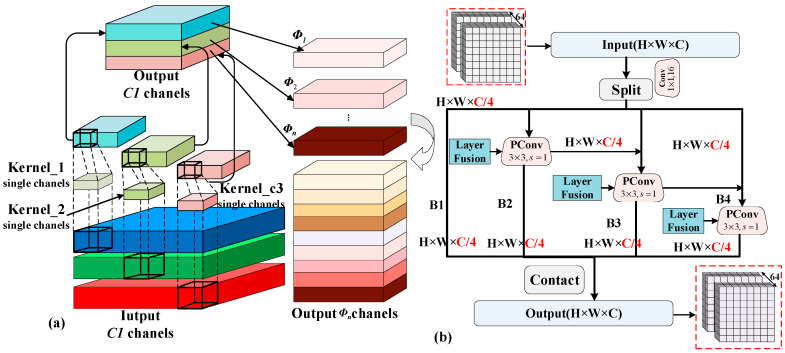
(**a**) The structure of the separable convolution and (**b**) MSFE Block (the input is split into chunks and connected via concatenation, the output gains a larger receptive field in the module, while the additional computational overhead remains negligible).

In this work, to solve (8), we design a cascaded restoration network (LJDD-Net) to recover $I_{B}$. Specifically, we develop four-stage architectures, as shown in Figure 4 and Figure 5, including pixel arrangement, shallow feature extraction, deep feature extraction, and a restoration layer. It [20] can be calculated by

(10) $$I_{B}(i,j)={\tilde{F}}_{\theta_{F}}(I_{A}\times M_{CFA}(i,j))$$

where ${\tilde{F}}_{\theta_{F}}$ denotes the proposed LJDD-Net to be trained with $\theta_{F}$ containing all the learnable parameters, $M_{CFA}$ denotes the Bayer domain with a size of H×W,i∈[0,H],j∈[0,W].

In the first stage of the model, it is generally necessary to apply downsampling through pixel rearrangement to obtain training pictures [28,48]. The first stage of the output can be expressed as

(11) $${\tilde{F}}_{0}(i,j)=W_{2\times 2}\left[M_{CFA}\times I_{A}\left(2i+(c\;\mathrm{mod}\;2),2j+\left\lfloor \frac{c}{2}\right\rfloor \right)\right]$$

where $W_{2\times 2}$ denotes the weight of downsampling, and $I_{A}$ is packed into a 4-channel feature map indexed by c ${\tilde{F}}_{0}\in {\mathbb{R}}^{\frac{h}{2}\times \frac{w}{2}\times 4}$ [28], $i\in [0,\frac{H}{2}-1],j\in [0,\frac{W}{2}-1]$, c∈[0,3].

In the second stage, to optimize the model for hardware deployment, the separable convolution structure is applied to significantly reduce memory access, as illustrated in Figure 5a. More specifically, a pointwise convolution is typically relevant after the depthwise convolution to enable feature fusion. For example, with a 3 × 3 kernel, memory access can be reduced by nearly 9×. Finally, the shallow feature stage produces feature maps with the same dimensions as the input. The convolution network constructed in this way can effectively reduce the number of parameters and make the network more lightweight. The output is expressed as

(12) $${\tilde{F}}_{1}(i,j)=\mathrm{ReLU}(\mathrm{PWC}(w_{pc1},\mathrm{DWC}(w_{dc3},{\tilde{F}}_{0}(i,j)))$$

where $w_{p1}$, $w_{dc3}$ refer to the weights of 1 × 1 and 3 × 3 convolutions, respectively.

In the third stage, the FasterNet block mainly consists of partial convolution (Pconv) as a basic operator, which is equivalent to a “T”-shaped convolution [26]. The mechanism is to keep the remaining channels unchanged instead of removing them from the feature map. Then, the 1 × 1 operator fuses the information from the previously excluded feature information dimensions. The output of the pre-stage in the third stage is ${\tilde{F}}_{2}\prime (i,j)\in {\mathbb{R}}^{h\times w\times 64}$, $w_{c_{p3\times 3}}$ is the weight of the Pconv, and it can be defined as

(13) $${\tilde{F}}_{2}\prime (i,j)=\mathrm{Pconv}(w_{c_{p3\times 3}},{\tilde{F}}_{1}(i,j))$$

### 3.3. Deep Feature Extraction and Restoration Layer

The pre-stage in the third stage is optimized by applying partial convolution to achieve low-cost memory access. In addition, we introduce a novel multi-scale feature extraction module (MSFE) at a granular level and increase the range of receptive fields for each network layer, which differs from the existing CNN multi-layer representation strength and improves the feature capability at a finer granularity [49].

Namely, ${{\tilde{F}}_{2}}^{\prime }$ is processed through a four-branch structure, where each branch applies a residual connection in a hierarchical manner. The output feature map, along with the other input feature maps, repeats this process across all four branches. In the post-stage, we apply a 3 × 3 partial convolution kernel as shown in Figure 5b. It can also be explained as

(14) $${\tilde{F}}_{2}(i,j)={\tilde{F}}_{2}\prime (i,j)⊗\sum \left(B1,B2,B3,B4\right)$$

where, ⊗ indicates a convolution operation.

(15) $$\begin{array}{l} B1={\mathrm{Pconv}}_{3\times 3}(w_{B1},{\tilde{F}}_{2}\prime (i,j)/4)\\ B2={\mathrm{Pconv}}_{3\times 3}(w_{B2},{\tilde{F}}_{2}\prime (i,j)/4)\\ B3=B2+{\mathrm{Pconv}}_{3\times 3}(w_{B3},{\tilde{F}}_{2}\prime (i,j)/4)\\ B4=B3+{\mathrm{Pconv}}_{3\times 3}(w_{B4},{\tilde{F}}_{2}\prime (i,j)/4) \end{array}$$

Then, ${\tilde{F}}_{2}(i,j)$ is connected with the four-group output through a 1 × 1 kernel, which fully integrates information from different branches. Finally, the upsampling is calculated through a deconvolution layer. The details of this operation can be defined as,

(16) $${\tilde{F}}_{3}(i,j)=\mathrm{Deconv}(w_{2\times 2},{\tilde{F}}_{2}(i,j))$$

where, ${\tilde{F}}_{3}(i,j)$ and $w_{2\times 2}$ denote the deconvolution and weight respectively.

### 3.4. Parameters and Analysis

Table 2 illustrates the parameters and FLOPs of LJDD-Net. By introducing partial convolution and depthwise separable convolution, the proposed model has 21.34 K parameters and 89.21 M FLOPs. In addition, compared with the CDM-CNN method first proposed by Gharbi et al. [28], the model parameters are reduced by 27.41× and 9.58×. It can be concluded that Pconv operations lead to a significant reduction in computational cost, facilitating hardware deployment.

**Figure 6:**
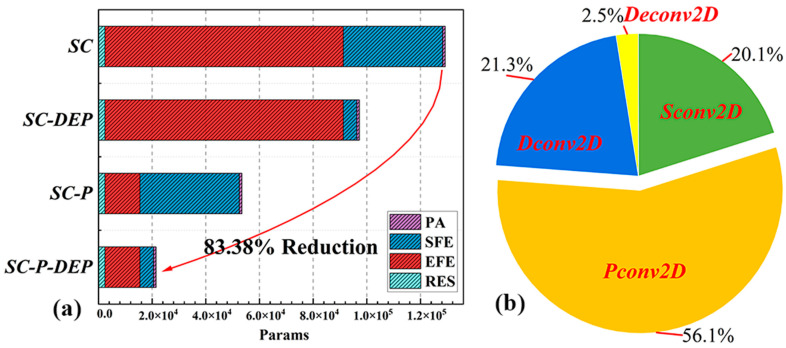
(**a**) The reduction in the number of parameters (SC: standard convolution; SC-P: only partial convolution; SC-DEP: only depthwise separable convolution; SC-P-DEP: both partial and depthwise separable convolution). (**b**) The distribution of MACs across each network stage, where PA, SFE, EFE, and RES correspond to the four stages of the network architecture.

Figure 6a shows the changes in the parameters of the proposed network structure under different convolution types. By applying partial convolution and depth-separable convolution, the parameters of the model are reduced by 83.38%. The parameters are the fewest in the PA stage, which is used to downsample and initialize the network structure layer. In addition, the parameters for shallow and deep feature extraction are less than one-fifth of those in previous works [3,32,50]. It can be concluded that most of the parameters are mainly concentrated in the enhancement feature extraction stage.

Figure 6b depicts the distribution of multiplication–addition arrays (MACs) in different convolutions. The LJDD-Net is mainly composed of partial convolutions, with a MAC ratio of 56.1%, standard convolutions of 20.1%, and depth-wise separable convolutions of 21.3%. In addition, the MAC of the reconstruction layer deconvolution is only 2.5%. In addition, compared with the standard convolutional solution, the parameters and MACs are reduced by 83.38% and 77.71%, respectively. Software-level results demonstrate a significant reduction in both parameters and computational cost (MACs) while maintaining high-quality visual restoration performance in the subsequent experiments.

## 4. Hardware Implementation and Optimization Strategy

In this section, we design the overall hardware implementation and optimization mechanism. In Section 4.1, we briefly introduce the hardware platform for the implementation of the Zynq UltraScale+. In Section 4.2, we elaborate on the details of the convolution calculation. In order to facilitate implementation on hardware, we introduce the layer fusion and quantization strategy in Section 4.3.

### 4.1. Data Flow and Module Implementation

**Figure 7:**
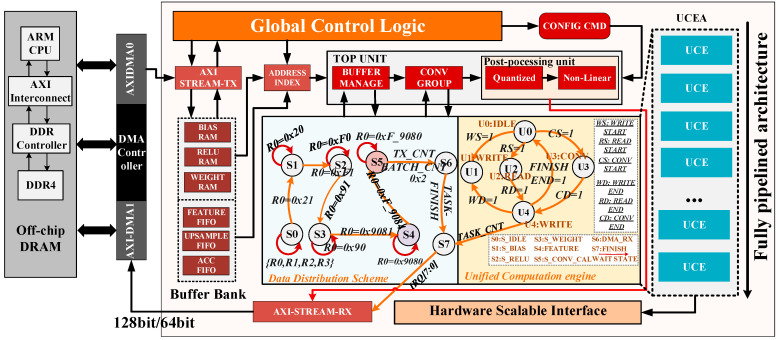
The overall architecture of the proposed design.

The overall hardware implementation is based on the Zynq UltraScale+ on-chip processing system, which consists of programmable logic (PL) and processor (PS). The hardware implementation is illustrated in Figure 7, mainly containing global control logic (GCL), a buffer bank (BB), a top module (TM), a Data Distribution Scheme (DDS), a unified computation engine (UCE), a post-processing unit (PPU), an address index module (AIM), direct memory access (DMA), AXI-STRAM-TX/RX, a unified computation engine array (UCEA), and a hardware scalable interface (HSI).

***GCL***: Compared with the conventional soft-core control approach (with an operating system)----, we adopt GCL as the top-level controller. The control logic is implemented through a register group based on the AXI-Lite bus, which manages and drives modules including UCEA, TM, DDS, UCE, and PPU. During the computation of SConv, DeConv, PConv, and DepConv layers, input data is transferred from off-chip memory to the BB through DMA. DMA can obtain data information such as the source address of the data, the number of input and output channels, and the feature map. Based on the proposed UCE calculation pattern, it can process multi-type convolutions of input patches while maintaining data arrangement consistency between input and output activations. By applying a fully pipelined architecture, the hardware design improves modularity and reduces development complexity. Specifically, the dimension of the input feature map channel is m, the output feature map channel is n, and the parallelism of UCEA is q, and it is set with two parameters m,n,q set to 8,8,8 and 16,16,16, respectively.

***DSS and UCE***： The DSS is mainly composed of the BB and AIU. The BB is partitioned into six buffer banks for data deployment. Weight, activate, and bias data are stored in BRAM, while upsampling, feature, and accumulation data are stored in FIFOs. The AIU performs read and write operations through BRAM address indexing and FIFO interfaces, respectively. Furthermore, data such as weights and activations only need to be loaded once. While computing intermediate results, the data is temporarily stored at specific addresses in DDR4. Specifically, Figure 7 depicts the 2×2 convolution operation with a step size of 2, Once the PS is to boot, while the register array, including {R0,R1,R2,R3} is initialized immediately, R0 is set *0x21* to load bias data, *0xF1* loads activate data, *0x91* loads weight data, *0x9081* loads the feature data. When *R0* is *0x9084*, the convolution calculation begins to work. When it is set to *0x2*, it is necessary to judge the numbers of the batch channel counter and transfer counter, whether the results of the UCE carry to the output or temporary output cache storage of the on-chip system. In addition, {R1,R2,R3} is mainly designed to manage the PPU module including the quantization parameters (scale and zero point) and AIM.

In addition, the UCE calculation module determines the completion of data read/write from DSS by checking the status of *(WS/WD)*, *(RS/RD)*, and *(CS/CD)*. The data is processed in batches through interrupt control (TASK-CNT). The wait state is to facilitate automatic status clearing, which is triggered by pulling the signal high for one cycle after a subtask completion. Once the previous layer’s computation finishes, the inferred pixel value is passed to the next layer’s calculation engine. In other words, the “batch by batch” computing mode is implemented. Upon collecting sufficient data, the UCEA module outputs the calculation results through the AXI-STREAM-RX interface, ensuring efficient data transmission.

***PPU***: The module performs non-linear functions. During quantization, multiplier operations are replaced by shift and rounding operations to reduce hardware computation cost. The data received from UCE is sent to the off-chip memory. Interrupt (IRQ) is executed multiple times to execute multiple batches of computing data. Through the HSI, the multi-parallelism (64/128bit) can be arbitrarily expanded to adapt to the limitations of different hardware platforms.

### 4.2. Details of Convolution Calculation

The convolution computation typically employs the Im2col + GEMM strategy [47]. In addition, high-level synthesis (HLS) generates redundant logic, which limits the utilization of on-chip resources and hinders the realization of high performance. This paper proposes a unified computation array, a flexible matrix construction method, and a padding mode with zero resource overhead. This method has the advantage of continuous addressing of feature maps and edge pixels. It also provides maximum design flexibility and supports fine-grained optimization.

(1) ***Unified computing engine***

**Figure 8:**
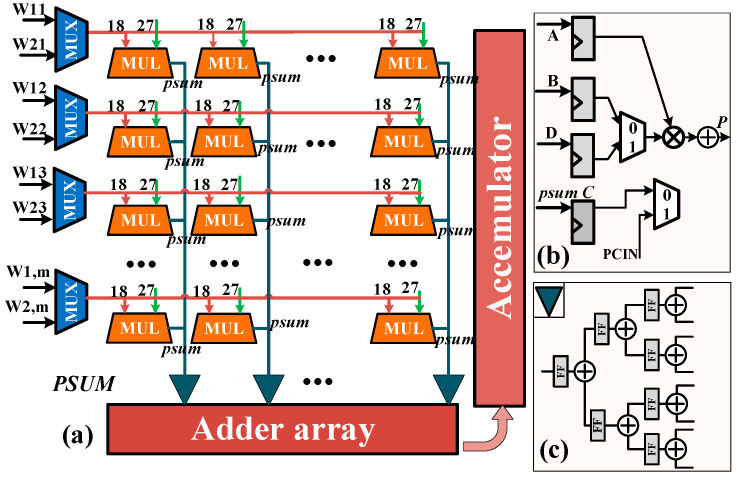
(**a**) Description of the UCE (the green line represents the input of the feature map), (**b**) schematic diagram of MUL internal structure, (**c**) three-stage pipeline addition tree. (A/B/C/D represent the input logic, FF represents the trigger, and has been added).

Figure 8a shows the calculation array structure (MUL) in the UCE, where $W_{a,b}$ represents the weight data of the *b*-th channel of the *a*-th convolution kernel and the *m*-th channel data of the input feature map ($IFM_{a}$). PSUM and psum represent the partial sum results from the previous and current cycles, respectively. Moreover, to leverage the internal resources of the MUL, an efficient data flow architecture and an MUL array structure with inter-channel cascading are proposed. Two convolution results are output through time-division multiplexing of an MUL, as illustrated in Figure 8b.

Specifically, the input feature map data is fixedly fed from port A, while the weight data for the two convolution kernels are provided through port B and port D, respectively. The two kernels reuse the input feature map. By configuring the MUL, its multiplier alternates between A × B and A × D operations in a ping-pong manner. Simultaneously, the adder alternates between accumulating the results of A × B and A × D, combining them with the previous channel results. MUL in the cascade receives the result of the previous channel through port C, while the remaining MULs cascade the results from the other previous channels through port PCIN. Finally, the psum results from the two channels are output by

(17) $$P=(A\times 2^{18}+D)\times B+C$$

In addition, a three-stage pipelined addition tree performs accumulation in the UCE array.

(2) ***Padding mechanism and construction matrix***

**Figure 9:**
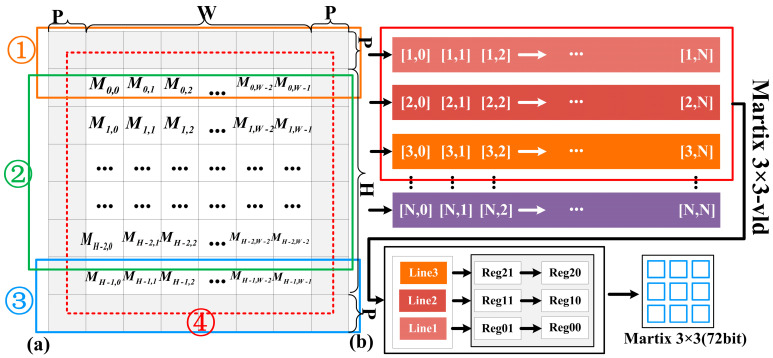
(**a**) Three padding strategies (① including the first line, ② excluding both the first and last lines, and ③ including the last line. ④ represents the padding pixel value). (**b**) Unified matrix construction mode.

Figure 9a illustrates the padding address translation and unified matrix construction scheme. The feature map stored in BB has a width of W and a height of H, and is zero-padded by P pixels along all borders. $M_{H,W}$ and ${\tilde{M}}_{H,W}$ represent the virtual address and physical address, respectively. When accessing the padding region, the address is zero, the invalid address signal is asserted, and a zero value is output to the UCE. When the valid BB data is accessed, $M_{H,W}$ must be translated into ${\tilde{M}}_{H,W}$, and the corresponding data is then forwarded to the UCE for calculation. Specifically, if Line < P or Line ≥ P + H, where Line indicates the current row in BB, the address corresponds to a padding region and no address translation is required. Otherwise, the conversion from virtual to physical address is defined by (17), and its simplified form is shown in (18). In addition, Figure 9a shows that the three padding modes are controlled by the {R0,R1,R2,R3} group in the implementation.

(18) $${\tilde{M}}_{H,W}=M_{H,W}-(Line\times (2P+W)+P)+(Line-P)\times W$$

(19) $${\tilde{M}}_{H,W}=M_{H,W}-P\times (2\times Line+1+W)$$

i.e.,

(20) $$(Line-P)\times W\leq {\tilde{M}}_{H,W}\leq (Line-P+1)\times W$$

Figure 9b demonstrates the unified construction matrix module. In contrast to a FIFO-based design with a fixed buffer size, we use a shift register array to construct the convolution. The shift register array can flexibly cope with different feature maps. In detail, by implementing it as a register array, we can construct any IFM, such as 3 × 3. In addition, the essence of separable convolution is the lack of an accumulation module, and the calculation of partial convolutions needs to focus on the separation and fusion of channel dimensions. In summary, based on the proposed memory access scheme, this paper achieves the “0” hardware resource overhead of the padding operation by modifying the address remapping mechanism.

(3) ***Timing diagrams of different convolution modules***

**Figure 10:**
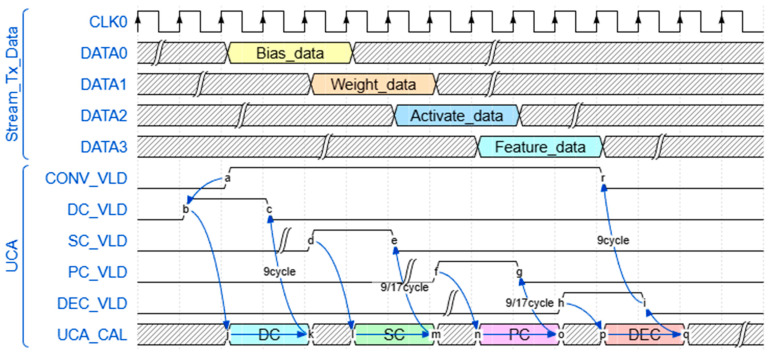
Timing diagram of data transmission and multi-type convolution calculation. In addition, (a)–(r) denote the signal edges, and the blue arrows indicate the clock cycles of the operator.

Figure 10 shows that the data is transmitted in sequence through AXI-Stream, mainly including weight data, feature map data, and activation data. For the 1 × 1 and 2 × 2 convolution operations, the conventional matrix construction is bypassed by reusing the 3 × 3 convolution calculation unit. At the same time, the multi-type kernels can directly access the corresponding data in the BB module through pipelined read and write operations. In addition, for skip-add operations (e.g., B1/B2/B3/B4 in the MSFE module), an address space needs to be temporarily stored for subsequent accumulation. Figure 10 also shows the timing diagram of various types of convolution calculations. To clarify, the latency is 9 (1 × 1, 2 × 2, 3 × 3 kernel), 17 (with padding) cycles towards DC/PC/SC/DEC.

### 4.3. Layer Fusion and Quantization

**Figure 11:**
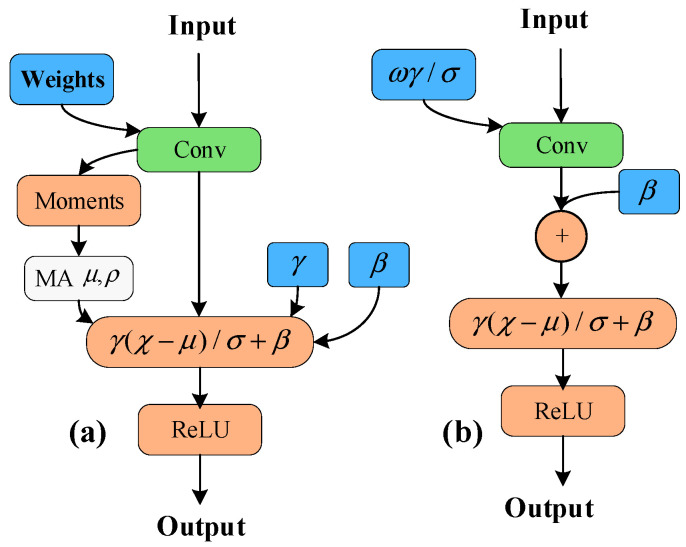
(**a**) Unfused network structure, (**b**) fusion strategy of conv. and BN layers.

When training the models, batch normalization (BN) layers can accelerate network convergence and control overfitting, thereby improving the generalization ability of the network. In order to reduce the data transmission between UCE and BB, in this paper, we adopt the BN folding method of QAT to fuse the BN layer with the convolution layer to reduce the calculations, as shown in Figure 11.

Assuming a network layer with input $x_{i}$ and the output after applying BN is $y_{i}$, it can be defined as,

(21) $$y_{i}=\gamma {\hat{x}}_{i}+\beta$$

where ${\hat{x}}_{i}=\frac{x_{i}-\mu_{B}}{\sqrt{\sigma_{B}^{2}+\varepsilon }}$, γ is the weight, β is the bias, $\mu_{B}$ is the mean, $\sigma_{B}^{2}$ is the variance, and ε is the constant. To fold BN into the weights and bias of the previous layer, applying the BN in the (20), it can be expressed as

(22) $$y_{i}=\gamma \frac{z_{i}-\mu_{B}}{\sqrt{\sigma_{B}^{2}+\varepsilon }}+\beta$$

i.e.,

(23) $$y_{i}=\gamma \frac{\omega x_{i}+b-\mu_{B}}{\sqrt{\sigma_{B}^{2}+\varepsilon }}+\beta$$

It can be concluded that the fusion of the operation is, $\omega_{fold}=\frac{\gamma \omega }{\sqrt{\sigma_{B}^{2}+\varepsilon }}$, $b_{fold}=\frac{\gamma (b-\mu_{B})}{\sqrt{\sigma_{B}^{2}+\varepsilon }}+\beta$.

Subsequently, the quantization module is applied after layer fusion, converting the model’s floating-point numbers into fixed-point representations [51], which can be explained as,

(24) $$q_{\mathrm{int}}=2^{-n}M_{0}\times \lambda_{\mathrm{int}}\approx (M_{0}\gg shift)\times \lambda_{\mathrm{int}}$$

where, $M_{0}$ is in the interval [0.5, 1) and n is a non-negative. $q_{\mathrm{int}}$ and $\lambda_{\mathrm{int}}$ denote the result of the quantization and the calculation of the intermediate integer, respectively. In this article, the model size is reduced from 257 KB to 88 KB by quantization. In addition, the nonlinear unit is implemented by the lookup table method.

## 5. Evaluation and Experimental Results

Section 5 evaluates the capabilities of the designed algorithm and the advantages of hardware implementation. Some recently published FPGA-based DM&DN methods are selected for comparison, such as traditional interpolation-based algorithms, machine learning methods, deep learning methods, etc. V-A introduces the evaluation indicators and experimental setup, V-B and V-C are a qualitative and quantitative comparison. V-D compares the resources of hardware implementations of different solutions.

### 5.1. Preliminary

We selected mainstream test sets, such as Kodak24 [52] McMaster [53],WATERLOO [54], Urban100 [55], DIV2K [56], and Flickr2K [57].To evaluate the quality of restored full-color high-resolution images, the color peak signal-to-noise ratio (CPSNR) and structural similarity (SSIM) indicators are commonly applied. For the denoising task, since the original dataset only consists of clean images, we synthesize noisy images at different levels by adding Gaussian noise with $\sigma \in \left\{5,10,15\right\}$. The overall architecture is designed with Verilog HDL. Finally, we leverage Vivado2022.2 and Vitis 2022.2 to synthesize and implement the RTL design on an FPGA device, Xilinx Zynq UltraScale + XCZU15EG Evaluation Board.

### 5.2. Training Strategy and Ablation Study

The model training details are as follows: The experiment is based on the WSL-Ubuntu-20.04 OS, the PyTorch 2.4.1 framework, the CUDA version is 12.1, the programming language version is Python 3.9, and the GPU is RTX 3090 (memory size is 24 GB). The CPU is Intel i7-10700K@2.60 GHz, and the DRAM is 32 GB. The Adam optimization method is used to update the trainable parameters, the initial learning rate is 0.0001, and the weight decay coefficient is $1\times 10^{-6}$. In addition, the batch size of the model loading data is set to 16, and the subprocesses is set to 16. Then, the training and testing datasets are selected from DIV2K [56] and Reference [28], with 79,935 and 19,960 pictures randomly sampled as training and test sets, respectively.

In addition, a 128 × 128 patch is applied during training. To enhance the dataset and improve sample diversity and robustness, random rotations of 90°, 180°, and 270° are applied with a probability of 0.5. Moreover, introducing Gaussian noise during training further enhances the model’s generalization to previously unseen data. During the training of LJDD-Net, the reverse gradient descent process of the training process, for the i-th group of training pairs $\left\{x^{\left(i\right)},y^{\left(i\right)}\right\}$, we define the loss function as the average loss by,

(25) $$l(\Theta )=\frac{1}{N_{b}}{\sum_{i=1}^{N_{b}}\left\|\varphi_{LJDD}(x^{i};\Theta )-y^{\left(i\right)}\right\|}_{2}^{2}$$

$\varphi_{LJDD}(\cdot )$ denotes the function of LJDD-Net, and Θ denotes the corresponding parameter set; $N_{b}$ is the batch size.

**Figure 12:**
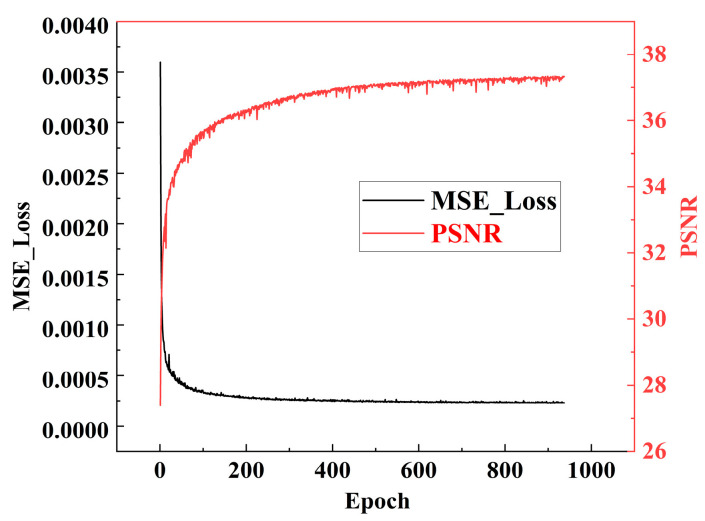
The trend of the PSNR and MSE curves during model training.

**Figure 13:**
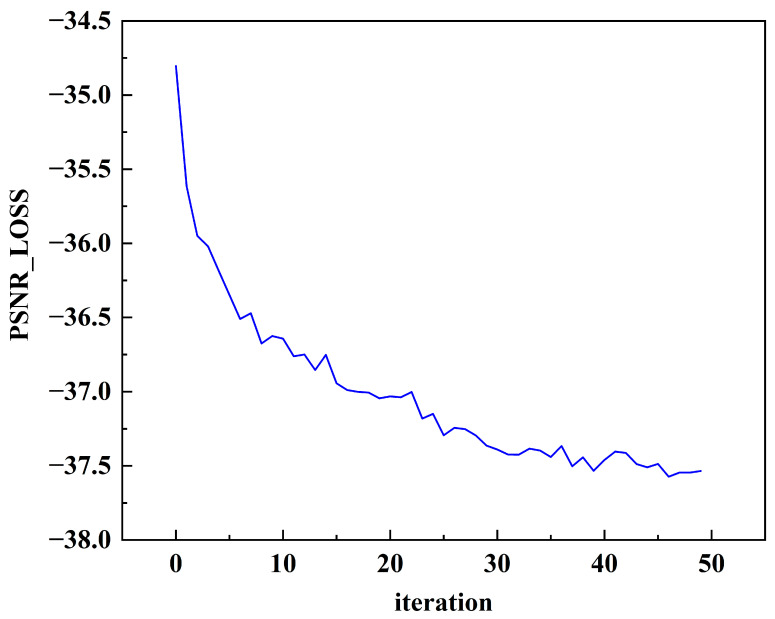
The PSNR−Loss in the (calibration + QAT) fine-tuning process.

The proposed algorithm benefits from the advantages of batch normalization and residual learning. The model rapidly decreases and nears convergence after approximately 400 epochs. Furthermore, the training time for the model is about two days. Upon reaching around 930 epochs of training, as shown in Figure 12, both the MSE and PSNR exhibit stabilizing trends. At this moment, the PSNR and MSE are approximately 37.5 dB and 0.0003, respectively. In addition, we adopt a calibration followed by a QAT strategy for evaluation. Specifically, PSNR is employed as the loss function, and a subset of the training dataset is used for testing. Figure 13 shows that the quantized model reaches performance comparable to the FP32 baseline after approximately 50 iterations of fine-tuning.

Table 3 represents the results of ablation experiments conducted on three different datasets with various noise levels. Yellow and green highlights indicate the best performance under the same conditions, while the results of our method are marked in red and blue fonts. The PSNR and SSIM values of the proposed LJDD-Net (*SC-DEP-P*) method differ from the best restoration results by 0.71 dB/0.68 dB/0.97 dB and 0.0198/0.0284/0.0559 at noise levels σ = 5, σ = 10, and σ = 15, respectively. From the comparison between *SC* and *SC-P*, it can be observed that introducing partial convolution improves SSIM under certain conditions. The PSNR difference remains within 1 dB, and the maximum difference in SSIM is approximately 0.05. Overall, the model proposed in this paper demonstrates strong potential for deployment on real-time edge-embedded platforms.

### 5.3. Quantitative and Qualitative Comparison

**Figure 14:**
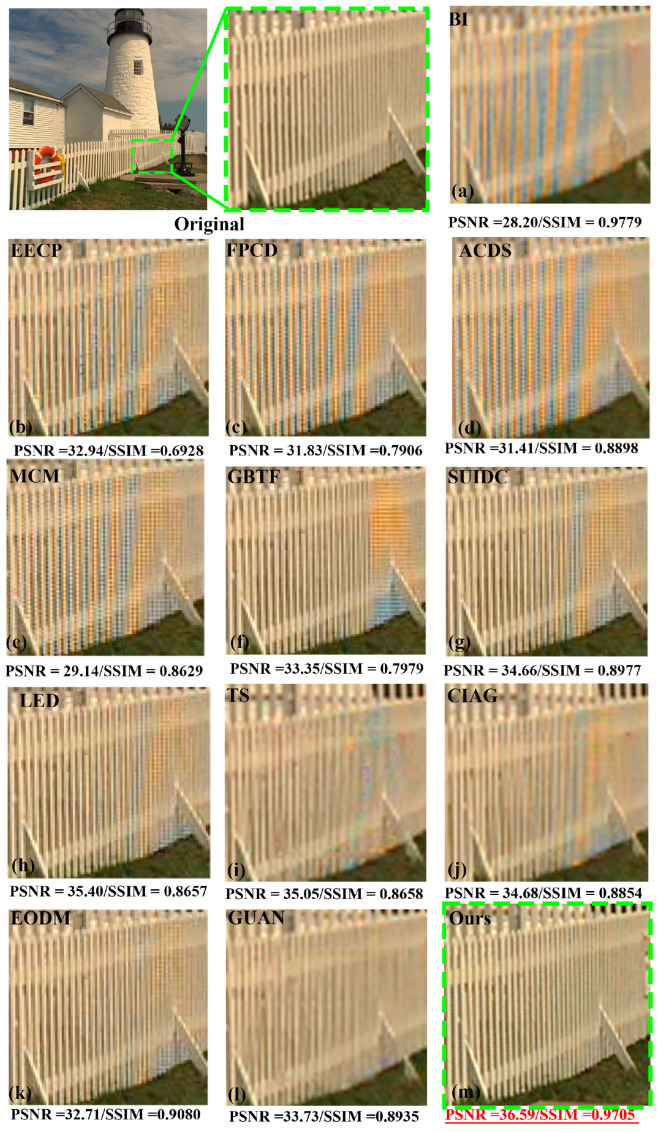
Results of traditional DM&DN methods (**a**–**h**,**j**,**k**) and model architectures (**i**,**l**,**m**) on Kodim24.

When comparing the performance of DM&DN methods, it is important to compare the presence of zipper noise and artifacts. Figure 14 shows the visual quality of our LJDD-Net results compared with other methods, including BI [40], EECP [58], FPCD [59], ACDS [60], MCM [61], SUIDC [10], LED [41], CIAG [45], EODM [37], GBTF [62], TS [44], and Guan [27] (where our results are shown in red and blue fonts). These methods will have false color artifacts, which are quite poor visual effects, both from the evaluation of objective indicators and subjective judgment.

As shown in Table 4, we compare the results with other references on different benchmarks. The average PSNR and SSIM of the L.R. and H.R. datasets are 36.8/0.9821, 34.87/0.9697, and 37.34/0.9854, 34.9/0.9699, respectively. In the hardware implementation, the results are 3.26% and 7.87% better than the current best processing results. Specifically, Guan [27] first proposed a deformable neural network model based on an FPGA acceleration system, but the model has no padding, and the edge texture restoration effect is still unsatisfactory. Our solution-based partial convolution can accurately insert the missing pixel values without any artifacts, achieving a better restoration effect. At the same time, compared with the deformable network structure, the complexity and number of parameters of the model have been further reduced. Compared with [27], PSNR and SSIM are improved by 2.36 and 0.0806, respectively.

**Figure 15:**
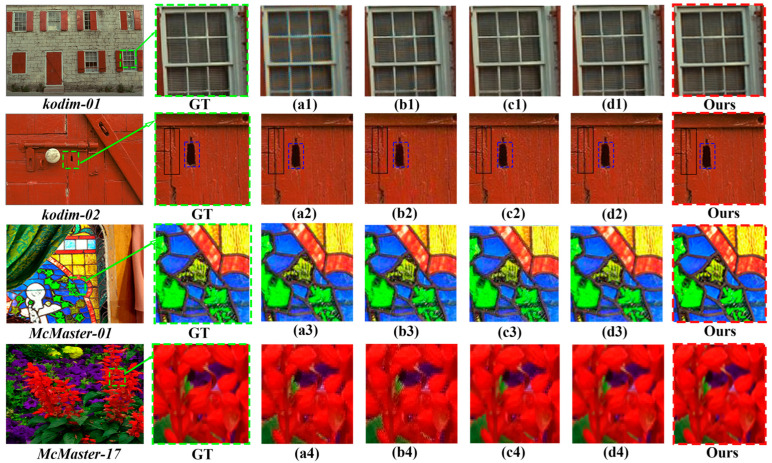
Comparisons of image reconstruction on different datasets: (**a1**–**a4**) CDM-CNN [28], (**b1**,**b2**) DDSN [29], (**b3**,**b4**) PyNET [34], (**c1**,**c2**) 3-STAGE-CNN [30], (**c3**,**c4**) NTSDCN [19], (**d1**,**d2**) HQDNN [7], (**d3**,**d4**) CDM-CNN [28], ours (LJDD-Net).

Then, we compare our method with only software-based implementations, as illustrated in Figure 15. For further evaluation, images with more complex scenes—such as Kodim01, Kodim02, McMaster01, and McMaster17—are selected from the Kodim24 and McMaster18 datasets. We compare some fine-grained details, such as Kodim01, and find that no false color or other reflection noise is generated on the windows. It can be concluded that the proposed method has excellent advantages in restoration quality and visual details, and our results have competitive advantages.

### 5.4. Hardware Implementation Comparison

Table 5 demonstrates the implementation results of the DM&DN system based on the CNN accelerator architecture on the Zynq UltraScale+ FPGA board. We compare the traditional algorithm, the model-based algorithm proposed in this paper, and several other image processing task accelerators. It can be observed that the traditional algorithm [39,45] consumes fewer resources and operates at a lower frequency, making it insufficient to ensure a real-time frame rate.

Computation efficiency [67] normalized efficiency by considering DSP conditions of different FPGA boards. The power consumption of the CPU and GPU was recorded by AIDA64. (In addition, both the CPU and GPU were warmed up for more reliable results.)

***(1). Comparison of Hardware Acceleration Solutions***: We proposed two different task parallelisms of 8 and 16, achieving 145.64 GOPS and 291.20 GOPS, respectively, which meet the requirements of edge-embedded devices. Compared with [44], the model proposed in this paper has a wide range of convolution kernel sizes and supports different strides. Compared with [27], when the parallelism is 16, the number of DSP macros is reduced by 49.31%, while the computational efficiency is improved by 8.70%. Moreover, the LUTs, FFs, and BRAM slices are also significantly reduced. When the task parallelism is 8, the computational efficiency increases by 2.09×, which is mainly due to the reuse of DSPs/PEs. Furthermore, by deploying partial convolution on UCEA, the number of multiplication and accumulation operations is significantly reduced. Once standard convolutions are fully implemented, the GOPS will be further improved. Moreover, compared with other hardware accelerator solutions [64,65,66] for image processing tasks, the proposed design achieves higher computing efficiency. Specifically, at parallelism levels of 8 and 16, the efficiency is improved by 3.50×, 3.69×, and 2.00×, and by 1.82×, 1.92×, and 1.04×, respectively. Furthermore, compared with [64,66], the energy efficiency is also enhanced, with improvements of 3.80×, 2.36×; 4.47×, and 2.78× at the two parallelism levels, respectively.

***(2). Comparison with CPU and GPU***: We evaluated our CPU- and GPU-based implementations on widely used platforms, namely an Intel Core i7-10700K processor and an NVIDIA RTX 3060 GPU. The LJDD-Net model was executed in the floating-point (FP32) format using the PyTorch framework, with 100 images randomly selected for testing. Compared with the two parallelization schemes, the proposed hardware accelerator achieves energy-efficiency improvements of 72.56× and 24.28× on the CPU platform, and 85.39× and 28.58× on the GPU platform, respectively, which fully demonstrates its advantages.

Table 6 shows the comparison of different convolution acceleration solutions and demonstrates the scalability of the proposed solution for various pixel processing tasks. Specifically, the proposed accelerator can flexibly and configurably support different convolution types, including SC, PC, DEP, and DEC, as well as a wide range of convolution kernels and strides. It can be concluded that the design is hardware-friendly for the DM&DN tasks and other image pixel processing applications on edge-embedded devices.

## 6. Conclusions

In this paper, we propose a joint DM&DN approach based on partial convolution to reduce computational cost. Experimental results show that LJDD-Net outperforms existing methods in terms of objective image quality metrics. We further present a unified and flexible multi-parallel hardware acceleration scheme. The scheme requires fewer hardware resources while achieving higher computational efficiency and a balance between speed and accuracy. In summary, we show positive advantages in both visual restoration and hardware performance. The proposed solution will be extended to more pixel processing tasks in the future. The research may benefit those who are planning to implement deep learning deployment on resource-limited embedded platforms.

## Figures and Tables

**Table 1:** Trends of vehicle cameras in autonomous driving [4].

Level	Computational Capability	Camera Count
L1	<1 TOPS	1
L2	>10 TOPS	5+
L3	>100 TOPS	8+
L4	>500 TOPS	10+
L5	>1000 TOPS	12+

**Table 2:** The input and output feature maps of each node and the number of parameters of the model.

Node	Input Feature Map	Output Feature Map	Params
1-0	[64, 3, 128, 128]	[64, 4, 64, 64]	52
1-1	[64, 4, 64, 64]	[64, 64, 64, 64]	2368
2-0	[64, 64, 64, 64]	[64, 64, 64, 64]	896
2-1	[64, 64, 64, 64]	[64, 64, 64, 64]	4160
3-0	[64, 64, 64, 64]	[64, 16, 64, 64]	2304
3-1	[64, 64, 64, 64]	[64, 64, 64, 64]	10,668
4-0	[64, 64, 64, 64]	[64, 12, 64, 64]	780
4-1	[64, 12, 64, 64]	[64, 6, 128, 128]	147
4-2	[64, 6, 128, 128]	[64, 3, 128, 128]	21

**Table 3:** Quantitative average results of image denoising with different noise levels σ ∈ {5,10,15} on Kodak24, McMaster, and DIV2K datasets.

Model PSNR/SSIM	Kodak24	McMaster	DIV2K
	σ = 5 σ = 10 σ = 15	σ = 5 σ = 10 σ = 15	σ = 5 σ = 10 σ = 15
*SC*	34.65/31.49/28.82	33.58/31.53/28.93	34.90/31.95/29.22
	0.9380/0.7756/0.7081	0.9417/0.9004/0.8014	0.9387/0.8928/0.8025
*SC-P*	34.55/31.48/28.77	33.55/31.19/28.90	34.90/31.92/29.18
	0.9393/0.8815/0.7889	0.9479/0.9033/0.8295	0.9462/0.8933/0.8021
*SC-DEP*	34.51/31.38/28.31	33.46/31.11/28.56	34.89/31.83/28.77
	0.9389/0.8742/0.7566	0.9454/0.8958/0.8000	0.9453/0.8845/0.7700
*SC-DEP-P*	**33.94/30.81/27.85**	**32.91/30.58/28.09**	**33.95/31.27/28.31**
	**0.9195/0.8581/0.7330**	**0.9217/0.8747/0.7796**	**0.9170/0.8631/0.7499**

**Table 4:** Comparison of peak signal-to-noise ratio and structural consistency parameters on different datasets.

Datasets		L.R.				H.R.			
		Kodak	McMaster	Waterloo	Average	Urban100	DIV2K	Flickr2K	Average
Method	PREC.	PSNR/SSIM	PSNR/SSIM	PSNR/SSIM	PSNR/SSIM	PSNR/SSIM	PSNR/SSIM	PSNR/SSIM	PSNR/SSIM
BI [63]	FP32	29.46/0.5546	33.49/0.5234	28.52/-	30.49/0.539	31.43/0.7453	33.28/0.6930	35.26/0.7784	33.32/0.7389
EECP [58]	/	33.90/-	29.08/-	28.62/0.5043	30.53/0.5043	30.52/0.6623	34.15/0.7970	-/-	32.34/0.7297
FPCD [59]	/	33.57/-	30.25/-	34.52/-	32.78/-	-/-	35.52/-	-/-	35.52/-
ACDS [60]	/	29.80/0.5043	30.04/0.4432	29.68/0.5234	29.84/0.4903	32.06/0.6856	34.69/0.9068	-/-	33.38/0.7947
CIAG [45]	FP32	37.36/0.9658	30.49/0.8604	29.08/0.9256	32.31/0.9173	32.54/0.8827	30.51/0.9021	35.87/0.8689	32.97/0.8846
SUIDC [10]	INT8	34.94/0.8302	32.60/0.9052	29.83/0.9120	32.46/0.8825	32.04/0.8522	35.26/0.9020	36.23/0.9256	34.51/0.8933
TS [44]	FIX	35.47/-	29.83/-	31.89/	32.40/	34.21/-	33.26/-	35.31/-	34.23/-
Zhou [46]	BOPs	32.24/0.9154	37.24/0.9794	29.92/0.8739	33.13/0.9229	31.16/0.8958	-/-		31.16/0.8958
Guan [27]	8,8 *	33.73/0.8935	-/-	-/-	33.73/0.8935	-/-	-/-	-/-	-/-
**LJDD**	**FP32**	**38.35/0.9831**	**35.76/0.9785**	**36.29/0.9847**	**36.8/0.9821**	**34.56/0.9860**	**38.44/0.9830**	**39.02/0.9871**	**37.34/0.9854**
LJDD *	8,16 *	**36.09/0.9741**	**34.16/0.9619**	**34.35/0.9731**	**34.87/0.9697**	**32.68/0.9676**	**35.98/0.9704**	**36.04/0.9716**	**34.9/0.9699**

In the resulting images of the proposed method and other DM&DN methods deployed on FPGA, “*” represents the QAT fine-tuning of the result based on the hardware scheme, and L.R. and H.R. [37] denote the restoration of the high-resolution and low-resolution datasets, respectively. PREC denotes the precision. The shadow region represents the method based on the model architecture.

**Table 5:** Comparison of conventional DM&DN hardware implementations and neural network accelerator solutions.

	[39] 20’	[45] 20’	[64] 21’	[65] 22’	[66] 23’	[44] 21’	[27] 22’	CPU	GPU	Ours ^1^	Ours ^2^
Tasks	Traditional DM&DN Algorithm		Other Acceleration Solutions			DM&DN Based on Models					
**Platform**	Zynq-7045	Zynq 7020	ZCU102	Intel Arria10	Arria10 GX 660	Virtex-7	Zynq UltraScale+	i7-10700K	RTX3060	ZynqUtraScale + XCZU15EG	
**Precision (W,A)**	fixed	INT8	Mixed	8bit-fixed	12-8fixed	INT16	INT8	FP32		8–16*fixed	
**Parallelism**	-	-	-	-	-	-	-	-	**-**	**8**	**16**
**Clock (MHz)**	101	120	100	200	225	120	250	4360	1777	**275**	
**DSP Used**	21	9	214	607	1082	81	2304	-	-	304	1168
**BRAM**	124 (22.75%)	12 (8.57%)	126.5	769 (36%)	216 (24%)	15 (1.46%)	544 (59.65%)	-	-	137 (18.41%)	322 (43.28%)
**Logic**	1136 (LUT) 1241 (FF)	8997 (LUT) 9933 (FF)	39.1k (LUT) /-(FF)	207k (ALM)	28.2k (LUTs 10.3%) 14k (FFs 2.6%)	1605 (LUT) 3741 (FF)	20.50k (LUT) 29.35k (FF)	-	-	18.00k (LUT 3.42%) 18.98k (2.78%)	51.60k (LUT 9.82%) 147.56k (FF 21.62%)
**Computation Efficiency** **(GOPS/DSP)**	-	-	0.137	0.13	0.24	-	0.23	-	**-**	**0.48 ^1^**	**0.25 ^2^**
**Power(W)**			3.5	-	16.51	-	-	80.8	38.66	**3.92 ^1^**	**6.66 ^2^**
**Energy** **Efficiency** **(GOPS/W)**			9.77	-	15.72	-	-	0.512	1.53	**37.15 ^1^**	**43.72 ^2^**
**PE/DSP** **Reuse**	-	-	N/N	N/N	Y/N	N/N	Y/N	-	-	**Y/Y**	
**Kernel Range/Stride**		-	K = 3	K = 3/K = 1	K = 3	K/S = 3/1	K = 1,3/S = 1	-	-	**K/S = Wide range**	

^1,2^ It represents the results of two hardware-level parallelisms. * represents the QAT fine-tuning.

**Table 6:** Comparison of operators supported by different hardware acceleration solutions.

Metric	SC	PC	DEP	DEC	Configurable
Park [44]	✓	✕	✕	✓	✕
Guan [27]	✓	✕	✕	✕	✕
Sun [64]	✓	✕	✕	✕	✕
Wu [65]	✓	✕	✓	✕	✕
Huang [66]	✓	✕	✓	✕	✕
**Ours ^1,2^**	**✓**	**✓**	**✓**	**✓**	**✓**

^1,2^ It represents the results of two hardware-level parallelisms.

## Data Availability

The data presented in this study are openly available in https://github.com/ChaofWang/Awesome-Super-Resolution/blob/master/dataset.md (accessed on 9 August 2024).

## References

[1] Jiang H., Tian Q., Farrell J., Wandell B.A. (2017). Learning the Image Processing Pipeline. IEEE Trans. Image Process..

[2] Buckler M., Jayasuriya S., Sampson A. Reconfiguring the Imaging Pipeline for Computer Vision. Proceedings of the 2017 IEEE International Conference on Computer Vision (ICCV).

[3] Menon D., Calvagno G. (2011). Color Image Demosaicking: An Overview. Signal Process. Image Commun..

[4] (2021). Information Technology—Requirements for Automotive-Grade Cameras.

[5] Yu K., Li Z., Peng Y., Loy C.C., Gu J. ReconfigISP: Reconfigurable Camera Image Processing Pipeline. Proceedings of the 2021 IEEE/CVF International Conference on Computer Vision (ICCV).

[6] Ma K., Gharbi M., Adams A., Kamil S., Li T.-M., Barnes C., Ragan-Kelley J. (2022). Searching for Fast Demosaicking Algorithms. ACM Trans. Graph..

[7] Wang S., Zhao M., Dou R., Yu S., Liu L., Wu N. (2021). A Compact High-Quality Image Demosaicking Neural Network for Edge-Computing Devices. Sensors.

[8] Ignatov A., Timofte R., Zhang Z., Liu M., Wang H., Zuo W., Zhang J., Zhang R., Peng Z., Ren S. AIM 2020 Challenge on Learned Image Signal Processing Pipeline. Proceedings of the European Conference on Computer Vision.

[9] Ehret T., Facciolo G. (2019). A Study of Two CNN Demosaicking Algorithms. Image Process. Line.

[10] Verma P., Meyer D.E., Xu H., Kuester F. Splatty- a Unified Image Demosaicing and Rectification Method. Proceedings of the IEEE/CVF Winter Conference on Applications of Computer Vision.

[11] Hegarty J., Brunhaver J., DeVito Z., Ragan-Kelley J., Cohen N., Bell S., Vasilyev A., Horowitz M., Hanrahan P. (2014). Darkroom: Compiling High-Level Image Processing Code into Hardware Pipelines. ACM Trans. Graph..

[12] Schwartz E., Giryes R., Bronstein A.M. (2019). DeepISP: Toward Learning an End-to-End Image Processing Pipeline. IEEE Trans. Image Process..

[13] Rawat W., Wang Z. (2017). Deep convolutional neural networks for image classification: A comprehensive review. Neural Comput..

[14] O’Shea K., Nash R. (2015). An Introduction to Convolutional Neural Networks. arXiv.

[15] Ketkar N., Moolayil J., Ketkar N., Moolayil J. (2021). Convolutional Neural Networks. Deep Learning with Python: Learn Best Practices of Deep Learning Models with PyTorch.

[16] Gu J., Wang Z., Kuen J., Ma L., Shahroudy A., Shuai B., Liu T., Wang X., Wang G., Cai J. (2018). Recent Advances in Convolutional Neural Networks. Pattern Recognit..

[17] Jin Q., Facciolo G., Morel J.-M. A Review of an Old Dilemma: Demosaicking First, or Denoising First?. Proceedings of the the IEEE/CVF Conference on Computer Vision and Pattern Recognition Workshops.

[18] Din S., Paul A., Ahmad A. (2020). Smart Embedded System Based on Demosaicking for Enhancement of Surveillance Systems. Comput. Electr. Eng..

[19] Wang Y., Yin S., Zhu S., Ma Z., Xiong R., Zeng B. (2021). NTSDCN: New Three-Stage Deep Convolutional Image Demosaicking Network. IEEE Trans. Circuits Syst. Video Technol..

[20] Song B., Zhou J., Chen X., Zhang S. (2023). Real-Scene Reflection Removal with RAW-RGB Image Pairs. IEEE Trans. Circuits Syst. Video Technol..

[21] Conde M.V., McDonagh S., Maggioni M., Leonardis A., Pérez-Pellitero E. Model-Based Image Signal Processors via Learnable Dictionaries. Proceedings of the AAAI Conference on Artificial Intelligence.

[22] Huang T., Wu F.F., Dong W., Shi G., Li X. Lightweight Deep Residue Learning for Joint Color Image Demosaicking and Denoising. Proceedings of the 2018 24th International Conference on Pattern Recognition (ICPR).

[23] Chollet F. Xception: Deep learning with depthwise separable convolutions. Proceedings of the IEEE Conference on Computer Vision and Pattern Recognition.

[24] Kaiser L., Gomez A.N., Chollet F. (2017). Depthwise separable convolutions for neural machine translation. arXiv.

[25] Guo J., Li Y., Lin W., Chen Y., Li J. (2018). Network decoupling: From regular to depthwise separable convolutions. arXiv.

[26] Chen J., Kao S., He H., Zhuo W., Wen S., Lee C.-H., Chan S.-H.G. Run, Don’t Walk: Chasing Higher FLOPS for Faster Neural Networks. Proceedings of the IEEE/CVF Conference on Computer Vision and Pattern Recognition.

[27] Guan J., Lai R., Lu Y., Li Y., Li H., Feng L., Yang Y., Gu L. (2022). Memory-Efficient Deformable Convolution Based Joint Denoising and Demosaicing for UHD Images. IEEE Trans. Circuits Syst. Video Technol..

[28] Gharbi M., Chaurasia G., Paris S., Durand F. (2016). Deep Joint Demosaicking and Denoising. ACM Trans. Graph..

[29] Khadidos A.O., Khadidos A.O., Khan F.Q., Tsaramirsis G., Ahmad A. (2021). Bayer Image Demosaicking and Denoising Based on Specialized Networks Using Deep Learning. Multimed. Syst..

[30] Cui K., Jin Z., Steinbach E. Color Image Demosaicking Using a 3-Stage Convolutional Neural Network Structure. Proceedings of the 2018 25th IEEE International Conference on Image Processing (ICIP).

[31] Kumar S.P.P., Peter K.J., Kingsly C.S. (2023). De-Noising and Demosaicking of Bayer Image Using Deep Convolutional Attention Residual Learning. Multimed. Tools Appl..

[32] Guo Y., Jin Q., Facciolo G., Zeng T., Morel J.-M. (2023). Joint Demosaicking and Denoising Benefits from a Two-Stage Training Strategy. J. Comput. Appl. Math..

[33] Ratnasingam S. (2019). Deep Camera: A Fully Convolutional Neural Network for Image Signal Processing.

[34] Cho M., Lee H., Je H., Kim K., Ryu D., No A. (2023). PyNET-Q×Q: An Efficient PyNET Variant for Q×Q Bayer Pattern Demosaicing in CMOS Image Sensors. IEEE Access.

[35] Liang Z., Cai J., Cao Z., Zhang L. (2021). CameraNet: A Two-Stage Framework for Effective Camera ISP Learning. IEEE Trans. Image Process..

[36] Yan N., Ouyang J. (2020). Channel-by-Channel Demosaicking Networks with Embedded Spectral Correlation. arXiv.

[37] Lien C.-Y., Yang F.-J., Chen P.-Y., Fang Y.-W. (2018). Efficient VLSI Architecture for Edge-Oriented Demosaicking. IEEE Trans. Circuits Syst. Video Technol..

[38] Liu J., Gao Y., Xiong X., Xu D., Zhu X., Fan Y. A Hardware Friendly Demosaicking Algorithm Based on Edge Sensing. Proceedings of the 2022 IEEE 16th International Conference on Solid-State & Integrated Circuit Technology (ICSICT).

[39] Zhuang Y., Li D. Real time bayer raw video projective transformation system using FPGA. Proceedings of the 2020 IEEE Computer Society Annual Symposium on VLSI (ISVLSI).

[40] Ramanath R., Snyder W.E., Bilbro G.L., Sander W.A. (2002). Demosaicking methods for bayer color arrays. J. Electron. Imaging.

[41] Niu Y., Ouyang J., Zuo W., Wang F. (2019). Low Cost Edge Sensing for High Quality Demosaicking. IEEE Trans. Image Process..

[42] Yang B., Wang D. (2019). An Efficient Adaptive Interpolation for Bayer CFA Demosaicking. Sens. Imaging.

[43] Uhm K.-H., Choi K., Jung S.-W., Ko S.-J. (2021). Image Compression-Aware Deep Camera ISP Network. IEEE Access.

[44] Park J.H., Cheol Kim M., Lee B.D., Hoon Sunwoo M. Implementation of CNN based demosaicking on FPGA. Proceedings of the 2021 18th International SoC Design Conference (ISOCC).

[45] Pan X.-Y., Li C.-C., Hao W., Xue Y.-F. (2020). FPGA Acceleration of Color Interpolation Algorithm Based on Gradient and Color Difference. Sens. Imaging.

[46] Zhou Z., Duan X., Han J. (2024). A Design Framework for Generating Energy-Efficient Accelerator on FPGA toward Low-Level Vision. IEEE Trans. VLSI Syst..

[47] Wu C.-T., Isikdogan L.F., Rao S., Nayak B., Gerasimow T., Sutic A., Ain-kedem L., Michael G. VisionISP: Repurposing the Image Signal Processor for Computer Vision Applications. Proceedings of the 2019 IEEE International Conference on Image Processing (ICIP).

[48] Chang K., Li H., Tan Y., Ding P.L.K., Li B. (2022). A Two-Stage Convolutional Neural Network for Joint Demosaicking and Super-Resolution. IEEE Trans. Circuits Syst. Video Technol..

[49] Gao S.-H., Cheng M.-M., Zhao K., Zhang X.-Y., Yang M.-H., Torr P. (2021). Res2Net: A New Multi-Scale Backbone Architecture. IEEE Trans. Pattern Anal. Mach. Intell..

[50] Ehret T., Davy A., Arias P., Facciolo G. Joint Demosaicking and Denoising by Fine-Tuning of Bursts of Raw Images. Proceedings of the 2019 IEEE/CVF International Conference on Computer Vision (ICCV).

[51] Jacob B., Kligys S., Chen B., Zhu M., Tang M., Howard A., Adam H., Kalenichenko D. Quantization and Training of Neural Networks for Efficient Integer-Arithmetic-Only Inference. Proceedings of the IEEE Conference on Computer Vision and Pattern Recognition.

[52] Martin D., Fowlkes C., Tal D., Malik J. A Database of Human Segmented Natural Images and Its Application to Evaluating Segmentation Algorithms and Measuring Ecological Statistics. Proceedings of the Eighth IEEE International Conference on Computer Vision, ICCV.

[53] Dubois E. (2005). Frequency-Domain Methods for Demosaicking of Bayer-Sampled Color Images. IEEE Signal Process. Lett..

[54] Ma K., Duanmu Z., Wu Q., Wang Z., Yong H., Li H., Zhang L. (2017). Waterloo Exploration Database: New Challenges for Image Quality Assessment Models. IEEE Trans. Image Process..

[55] Huang J.-B., Singh A., Ahuja N. Single Image Super-Resolution from Transformed Self-Exemplars. Proceedings of the IEEE Conference on Computer Vision and Pattern Recognition.

[56] Agustsson E., Timofte R. NTIRE 2017 Challenge on Single Image Super-Resolution: Dataset and Study. Proceedings of the IEEE Conference on Computer Vision and Pattern Recognition Workshops.

[57] Lim B., Son S., Kim H., Nah S., Lee K.M. Enhanced Deep Residual Networks for Single Image Super-Resolution. Proceedings of the IEEE Conference on Computer Vision and Pattern Recognition Workshops.

[58] Chen S.-L., Ma E.-D. (2014). VLSI Implementation of an Adaptive Edge-Enhanced Color Interpolation Processor for Real-Time Video Applications. IEEE Trans. Circuits Syst. Video Technol..

[59] Chen S.-L., Chang H.-R., Lin T.-L. (2014). Ultra-low-cost Colour Demosaicking VLSI Design for Real-time Video Applications. Electron. Lett..

[60] Visvanathan V., Mohanty N., Ramanathan S. (1993). An Area-Efficient Systolic Architecture for Real-Time VLSI Finite Impulse Response Filters. Proceedings of the Sixth International Conference on VLSI Design.

[61] Hirakawa K., Parks T.W. Adaptive homogeneity-directed demosaicing algorithm. Proceedings of the 2003 International Conference on Image Processing (Cat. No.03CH37429).

[62] Pekkucuksen I., Altunbasak Y. Gradient Based Threshold Free Color Filter Array Interpolation. Proceedings of the 2010 IEEE International Conference on Image Processing.

[63] Jensen K., Anastassiou D. (1995). Subpixel Edge Localization and the Interpolation of Still Images. IEEE Trans. Image Process..

[64] Sun M., Li Z., Lu A., Li Y., Chang S.-E., Ma X., Lin X., Fang Z. FILM-QNN: Efficient FPGA Acceleration of Deep Neural Networks with Intra-Layer, Mixed-Precision Quantization. Proceedings of the 2022 ACM/SIGDA International Symposium on Field-Programmable Gate Arrays.

[65] Wu X., Ma Y., Wang M., Wang Z. (2022). A Flexible and Efficient FPGA Accelerator for Various Large-Scale and Lightweight CNNs. IEEE Trans. Circuits Syst. I Regul. Pap..

[66] Huang J., Liu X., Guo T., Zhao Z. (2023). A High-Performance FPGA-Based Depthwise Separable Convolution Accelerator. Electronics.

[67] Mao W., Su Z., Luo J., Wang Z. (2023). A Unified Acceleration Solution Based on Deformable Network for Image Pixel Processing. IEEE Trans. Circuits Syst. II Express Briefs.

